# Prize Questions for 1801, &c.

**Published:** 1801-04

**Authors:** 


### *Prize Questions for* 1801, *&c.*

THE anniverfary meeting of the Medical Society of London, was held at their houfe in Bolt Court, Fleet Street, on Monday, the 9th of March, when the Officers and Council for the year enfuing were elected; after which the annual Oration was delivered by Mr. Chamberlaine, furgeon, who gave a concife hiftory of the Society from its inftitution, and made fuitable and well deferved panegyrics on its founders, particularly on the refpectable phyfician who has been its greateft benefactor.

The orator then indulged his talents in beftowing the well merited praife on thofe members who had diftinguifhed themfelves by their improvements in the practice of Medicine and Surgery. He, however, laid it down as a rule not to be deviated from, that the name of no *Fellow* of the Society ought to be mentioned, as all might be fuppofed to be *prefent.* This circumftance led him, when enumerating the *correfponding members* who had eminently contributed to the advancement of medical knowledge, to omit the name of Dr. Jenner who was prefent, and whom Mr. C. believed to be a Fellow. The omiffion however was amply compenfated for in the courfe of the evening, Mr. C. having been informed of his miftake.— The annihilator of the Small-pox was drank among the principal benefactors of the human race.

Mr. C. alfo adverted to the act of parliament lately paffed for taking an account of the population of this country, which he confidered as likely to produce confiderable advantage, not only to the Science of Medicine, but alfo to that branch of the mathematics which is employed in calculating the chances of furvivorfhip, and the duration of life, derived from the Bills of Mortality; which latter will, no doubt, in future, be placed upon a more regular and certain fyftem than is now practifed. After many judicious obfervations upon the extreme inaccuracy and abfurdity of thefe Bills, which fatisfactorily evinced the neceffity of a reform, he adverted to the mifchief produced by the aftonifhing increafe of the fale of patent and other empiric medicines, and, in order to fhew how much more the ufe of thofe injurious compofitions prevail in South than in North Britain, he obferved that the net amount of ftamp duties on thofe articles paid in the former, in one year, was not far fhort of £. 14,000, while in the latter it produced fomething under £. 50, and thefe exclufive of the duty upon advertifements for the fame.

The Prefident then declared the adjudications of the Fothergillian, or gold medal, and alfo of the filver medal annually prefented by the Society; the former of which was delivered to Dr. Bouttatz, a Ruffian phyfician, for his Effay on the Medical Ufes of Phofphorus; the latter was adjudged to Dr. Jofeph Adams, of Madeira, a correfponding member of the Society, for his paper read before them on the Framboefia Guinenfis, an interefting account of a difeafe but little underftood in this country, although common in tropical climates.

The prize fubjects for the year 1801 were then announced, viz.

1. The caufe of the heat in fevers.

2. The medicinal effects of arfenic externally and internally applied.

3. The diftinguifhing characters of Scrophula in its various ftages, and in different parts of the body.

4. The caufes and treatment of deafnefs.

5. One or more difeafes, not generally known, peculiar to artificers in any branch of manufactures.

The Gold Medal, value ten guineas, will be given for the beft anfwer to any one of the above fubjects, agreeably to the following regulations:

1. Each Differtation fhall be delivered to the Secretary, in the Latin, Englifh, or French Language, on or before the firft day of November, 1801.

2. With each Differtation fhall be delivered a fealed packet with fome motto or device on the outfide; and within, the Author's name and defignation; and the fame motto or device fhall be put upon the Differtation, that the Society may know how to addrefs the fuccefsful candidate.

3. No paper with the name of the Author affixed can be received; and if the author of any paper fhall difcover himfelf to the Council, or to any member thereof, fuch paper fhall be excluded from all competition for the medal.

4. All the Differtations, the fuccefsful one excepted, fhall be returned, if defired, with the fealed packets unopened.

The Society continue to offer two filver medals annually; one to the author of the beft Effay, read before the Society within the year, by a Fellow, that is, any Member who refides within feven miles of London; the fecond to the beft Effay by any other perfon.

---

*The following is a List of the Officers and Committees forming the Council.*

PRESIDENT.

Dr. James Sims.

TREASURER.
Dr. Walker.

LIBRARIAN.

LIBRARIAN.

1 Mr. J. Hurlock.

SECRETARIES.
1 Mr. Field
2 Mr. J. H. Hooper.

SECRETARY FOR FOREIGN CORRESPONDENCE.

1 Dr. R. Hooper.

### COMMITTEES.

I. THEORY and PRACTICE of PHYSIC.
1 Dr. Sir J. M. Hayes, Bart.
2 Dr. Lettfom
3 Dr. Hulme
4 Dr. Saunders
5 Dr. Bradley.

II. ANATOMY and PHYSIOLOGY.
1 Dr. Relph
2 Dr. Fryer
3 Mr. Abernethy
4 Mr. Coleman
5 Mr. Mackartney.

III. SURGERY.
1 Mr. Ford
2 Mr. Norris
3 Mr. Ware
4 Mr. Forfter
5 Mr. Blair.

IV. MIDWIFERY.
1 Dr. John Sims
2 Dr. Haighton
3 Dr. Petch
4 Mr. Webb
5 Mr. Ring.

V. MATERIA MEDICA and PHARMACY.
1 Dr. Yellowly
2 Mr. Jackfon
3 Mr. Kilpatrick
4 Mr. Jeaffrefon
5 Mr. Mackinder.

VI. BOTANY and NATURAL HISTORY.
1 Dr. Bancroft
2 Dr. Thornton
3 Mr. Chamberlaine
4 Mr. Whately
5 Mr. Strong.

VII. NATURAL PHILOSOPHY and CHEMISTRY.
1 Dr. Babington
2 Dr. Elliot
3 Dr. Garnett
4 Mr. Parkinfon
5 Mr. Seaton

REGISTER.
Mr. Wheeler.

ANNIVERSARY ORATION FOR NEXT YEAR, Dr. Lowdell.

